# Risk factors for postoperative pneumonia in patients undergoing hip fracture surgery: a systematic review and meta-analysis

**DOI:** 10.1186/s12891-022-05497-1

**Published:** 2022-06-08

**Authors:** Seung-Beom Han, Sang-Bum Kim, Kyun-Ho Shin

**Affiliations:** 1 Department of Orthopedic Surgery, Anam Hospital, Korea University College of Medicine, Seoul, South Korea; 2 Joint Center, Inbone Hospital, Paju-si, Gyeonggi-do South Korea; 3 Department of Orthopedic Surgery, Nanoori Hospital (Incheon), 156, Jangje-ro, Bupyeong-gu, Incheon, 21353 Republic of Korea

**Keywords:** Pneumonia, Hip fracture, Hip surgery, Postoperative complications, Predictors, Systematic reviews, Meta-analysis

## Abstract

**Background:**

Postoperative pneumonia (POP) is a devastating complication that can frequently occur after hip fracture surgery. This study aimed to quantitatively and comprehensively summarize the risk factors for POP following hip fracture surgery.

**Methods:**

PubMed, Embase, and Cochrane Library were systematically searched for studies assessing risk factors for POP following hip fracture surgery. The pooled odds ratio (OR) and standardized mean difference (SMD) between patients with and without POP were calculated. Evidence was assessed using the Newcastle–Ottawa scale.

**Results:**

Ten studies including 37,130 patients with hip fractures were selected. POP occurred in 1768 cases with an accumulated incidence of 7.8% (95% confidence interval [CI]: 0.061–0.094). Advanced age (SMD: 0.50, 95% CI: 0.10–0.90), male sex (OR: 1.50, 95% CI: 1.12–2.01), American Society of Anesthesiologists physical status scale ≥3 (OR: 3.17, 95% CI: 1.25–8.05), chronic obstructive pulmonary disease (OR: 2.05, 95% CI: 1.43–2.94), coronary heart disease (OR: 1.82, 95% CI: 1.27–2.60), arrhythmia (OR: 1.49, 95% CI: 1.04–2.15), congestive heart failure (OR: 1.41, 95% CI: 1.14–1.75), chronic kidney disease (OR: 2.09, 95% CI: 1.28–3.41), and cerebrovascular accident (OR: 2.14, 95% CI: 1.60–2.85) were risk factors for POP. Hemoglobin (SMD: -0.14, 95% CI: − 0.25 to − 0.03), albumin (SMD: -0.97, 95% CI: − 1.54–-0.41), blood urea nitrogen (SMD: 0.20, 95% CI: 0.03–0.37), alanine aminotransferase (SMD: 0.27, 95% CI: 0.10–0.44), arterial oxygen pressure (SMD: -0.49, 95% CI: − 0.71–-0.27), time from injury to surgery (SMD: 0.13, 95% CI: 0.08–0.17), and surgery within 48 h (OR: 3.74, 95% CI: 2.40–5.85) were associated with the development of POP.

**Conclusion:**

Patients with the aforementioned risk factors should be identified preoperatively, and related prophylaxis strategies should be implemented to prevent POP following hip fracture surgery.

**Supplementary Information:**

The online version contains supplementary material available at 10.1186/s12891-022-05497-1.

## Background

Hip fractures are a major health problem and the number of hip fractures is expected to increase by approximately 2% annually over the next 30 years [1]. Hip fractures are associated with increased risk of morbidity and mortality [1–3]. Furthermore, the coronavirus disease pandemic, has forced an unprecedented period of challenge for the management of patients with hip fractures [4].

Postoperative pneumonia (POP) is a devastating complication that can occur after hip fracture surgery [5, 6]. However, few studies have been performed to elucidate this complication and investigate patients with hip fracture and POP. The incidence of hip fracture-related pneumonia has been reported to range from 4 to 15% [7–9]. Evidence has shown that POP is associated with various predisposing factors, including older age, male sex, multiple medical comorbidities, and hypoalbuminemia [7–14].

With the progress in medical technologies and aftercare of patients, clinicians are increasingly focusing on the prevention and treatment of POP. To medically optimize patients and provide better perioperative care, identifying various potential risk factors is important for POP. To the best of our knowledge, no formal systematic review and meta-analysis has investigated and summarized the risk factors for POP following hip fracture surgery. Therefore, this meta-analysis aimed to summarize the risk factors for the development of POP in patients undergoing hip fracture surgery. The results of this study are potentially beneficial for clinicians to identify high-risk patients and help prevent postoperative POP following hip fracture surgery.

## Methods

This study followed the Preferred Reporting Items for Systematic Reviews and Meta-Analyses (PRISMA) guidelines [15]. Patient consent and ethical approval were not required because this study was a meta-analysis of published studies. Two authors (KHS and SBH) independently searched and reviewed the literature, assessed the quality, and extracted data. Disagreements were resolved through discussions or negotiations with a third independent author (SBK). Inter-reviewer reliability was assessed by study screening and selection, quality assessment, data extraction, and result pooling using the kappa statistic (κ). The κ value for the data extraction ranged from 0.88 to 1.00.

### Search strategy

MEDLINE/PubMed, Cochrane Central Register of Controlled Trials, and EMBASE were exhaustively searched to identify original studies that included patients with hip fracture with POP published before January 4, 2022. The search terms, Medical Subject Headings terms, and their combinations searched in the title/abstract field of the search engines were as follows: “hip,” “fracture,” “hip fractures,” “pneumonia,” “lower respiratory tract infection,” “pulmonary infection,” “factor,” “risk,” and “predictor.” No other restrictions, including language, were applied. The references of the selected articles were also reviewed to identify relevant articles.

### Eligibility criteria and study selection

**Fig. 1:**
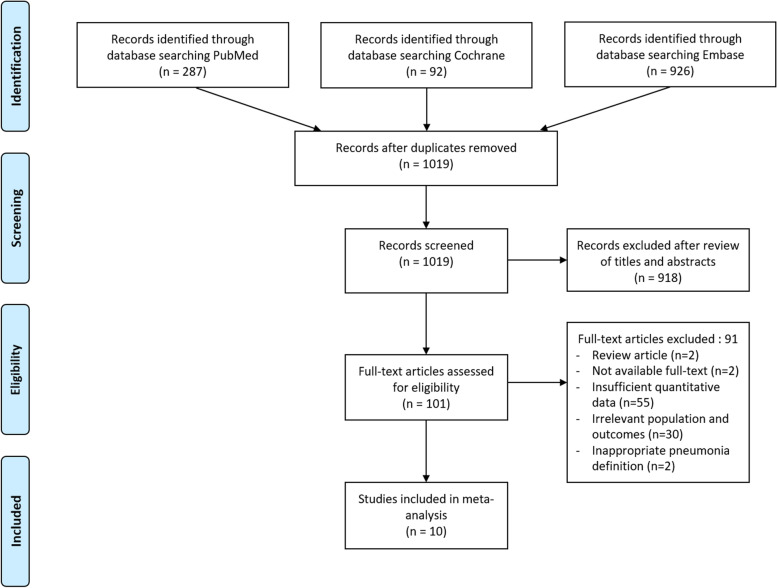
PRISMA flow diagram. PRISMA, Preferred Reporting Items for Systematic Reviews and Meta-Analyses

Two independent authors (KHS and SBH) screened all the titles and abstracts. Initially selected articles were further reviewed for inclusion according to the following inclusion criteria: (1) Cohort and case-control studies if they reported analyses of the predictors of POP in patients undergoing hip fracture surgery. (2) POP occurred after hip fracture surgery and recurited patients without pneumonia at baseline, (3) comparison between patients with POP as the case group and patients without POP as the control group; (4) accessible full-text articles; and (5) studies reporting sufficient information to extract and calculate relevant standardized mean difference (SMD) or odds ratio (OR) with 95% confidence interval (CI). The specific reasons for the excluded articles are shown in Fig. 1.

### Data extraction

Two independent authors extracted data from the eligible studies (KHS and SBH). Disagreements were resolved through discussion and consensus with the third author (SBK). Data were extracted according to the following descriptive information: (1) study characteristics, including the name of the first author, year of publication, study country, and study design; (2) patient demographics, such as the number of patients with or without POP, age, sex, and the incidence of POP; (3) significant risk factors for POP; and (4) number of citations for each potential risk factor for POP after hip fracture surgery.

### Quality assessment

The methodological quality of each included study was evaluated using the Newcastle–Ottawa scale (NOS) [16]. The scale includes selection, comparability, and outcome domains. The selection domain has four categories; comparability domain, two categories; and outcome domain, three categories. A study was awarded a maximum of one star for each category in the selection and outcome domains. A maximum of two stars was assigned to the comparability domain.

### Statistical analyses

ORs or SMDs with corresponding 95% CIs were estimated and pooled across studies to assess the association between POP and various potential risk factors. A meta-analysis was performed for each factor (*n* ≥ 2), which was presented as an effect size of the 95% CI. The adjusted data were used maximally when available. The inconsistency index (I^2^) was determined, and a χ^2^-based test of homogeneity was performed. If I^2^ was < 50%, the fixed-effects model (Mantel–Haenszel method) was used due to low heterogeneity. I^2^ ≥ 50% was considered a significant heterogeneity. The random-effects model (DerSimonian–Laird method) was used, and a “leave-one-out” sensitivity analysis was performed by sequentially deleting one study to determine the source of heterogeneity [17]. After excluding each study, an analysis was performed to determine the existence of heterogeneity. When 10 or more studies were included, a small study publication bias was assessed using funnel plot analysis. The significance level was set at *p* <  0.05. All statistical analyses were performed using RStudio v.1.0.143 (RStudio Inc., Boston, MA, USA).

## Results

### Search results

Figure 1 shows a detailed summary of the study’s identification and selection process. A total of 1305 articles were identified after the initial search. After eliminating 286 duplicates and 918 ineligible articles based on titles and abstracts, the full text of 101 articles were reviewed. After excluding 91 articles without information of inclusion criteria, 10 articles [7–14, 18, 19] were finally selected for the meta-analysis.

### Study characteristics

The characteristics of the included studies of POP are shown in Table 1. All studies were published in English and from 2016 onwards. All the included studies were retrospective cohort studies. A total of 1768 patients with hip fracture had POP and 35,362 patients without POP. The risk factors of POP reported in individual studies are summarized in Table 1.

**Table 1:** Characteristics of the included studies

			Sample size (n)			Age (years)		Male sex (%)			
First author (year)	Country	Study design	Total	Pneumonia	No pneumonia	Pneumonia	No pneumonia	Pneumonia	No pneumonia	Incidence (%)	Significant factors
Lv et al. 2016 [8]	China	Retrospective cohort study	1429	70	1359	Median 82	Median 74	22 (31.4)	575 (42.3)	70/1429 (4.9)	Age, male sex, fracture type, number of comorbidities, ASA ≥ 3, surgical type, preoperative hypoalbuminemia, high Cr, high RDW, preoperative mechanical ventilation
Bohl et al. 2018 [7]	USA	Retrospective cohort study	29,377	1191	28,186	NA	NA	NA	NA	1911/28,186 (4.1)	Age, male sex, COPD, low BMI, CHF, dyspnea on exertion, functional status, anemia
Chang et al. 2018 [10]	China	Retrospective cohort study	240	25	215	NA	NA	9 (36.0)	68 (31.6)	15/240 (6.3)	Age, CVA, cancer, low platelet, high blood glucose
Wang et al. 2019 [12]	China	Retrospective cohort study	720	54	666	82.3	77.5	20 (37.0)	27 (41.0)	54/720 (7.5)	COPD, CVA, preoperative hypoalbuminemia, time from injury to surgery
Salarbaks et al. 2020 [9]	Netherlands	Retrospective cohort study	407	62	345	Median 84	Median 83	29 (46.8)	98 (28.4)	62/407 (15.2)	Male sex, COPD
Shin et al. 2020 [11]	South Korea	Retrospective cohort study	1155	59	1096	83.1	77.9	21 (35.6)	295 (26.9)	59/1155 (5.1)	Age, cardiovascular disease, early postoperative hypoalbuminemia
Wang et al. 2020 [19]	China	Retrospective cohort study	293	33	260	84.5	85.1	20 (60.6)	76 (29.2)	33/293 (11.3)	Male sex, smoking, preoperative hypoalbuminemia, low arterial oxygen saturation
Xiang et al. 2020 [13]	China	Retrospective cohort study	1113	166	947	86.4	78.8	53 (31.9)	331 (35.0)	166/1113 (14.9)	Low BMI, preoperative hypoalbuminemia, high CRP, functional status, time from injury to surgery
Zhao et al. 2020 [14]	China	Retrospective cohort study	1495	53	1442	NA	NA	28 (52.8)	483 (33.5)	53/1495 (3.5)	Age, male sex, chronic respiratory disease, liver disease, urinary tract infection, high CK-MB, high BNP, high D-dimer
Ji et al. 2021 [18]	China	Retrospective cohort study	901	55	846	81.6	78.5	23 (41.8)	280 (33.1)	55/901 (6.1)	Age, COPD, CVA, hypoxemia, time from injury to surgery

*ASA* American Society of Anesthesiologists physical status, *Cr* Creatinine, *RDW* Red blood cell distribution width, *COPD* Chronic obstructive pulmonary disease, *BMI* Body mass index, *CHF* Congestive heart failure, *CVA* Cerebrovascular accident, *CRP* C-reactive protein, *CK-MB* creatine kinase MB, *BNP* B-type natriuretic peptide

### Risk of bias analysis

The risk of bias assessment of the included studies is summarized in Table 2. The NOS scores of the selected studies ranged from 8 to 9. Methods of the cohort selection and outcome assessment were clearly stated in all studies. Most studies excluded persons with pneumonia preoperatively. Most studies accounted for confounding factors using standard statistical regression techniques.

**Table 2:** Quality assessment of included studies

First author (year)	Selection				Comparability		Outcomes		
	Representativeness of the exposed cohort	Selection of the nonexposed cohort	Ascertainment of exposure	Demonstration that the outcome of interest was not present at the start of the study	Controlled for age and comorbidities	Controlled for any additional factors	Assessment of outcomes	Sufficient follow-up	Adequacy of follow-up
Lv et al. 2016 [8]	★	★	★	★	★	★	★	★	★
Bohl et al. 2018 [7]	★	★	★	★	★	★	★	★	★
Chang et al. 2018 [10]	★	★	★		★	★	★	★	★
Wang et al. 2019 [12]	★	★	★	★	★	★	★	★	★
Salarbaks et al. 2020 [9]	★	★	★	★	★	★	★		★
Shin et al. 2020 [11]	★	★	★	★	★	★	★		★
Wang et al. 2020 [19]	★	★	★	★	★	★	★		★
Xiang et al. 2020 [13]	★	★	★	★	★	★	★		★
Zhao et al. 2020 [14]	★	★	★	★	★	★	★		★
Ji et al. 2021 [18]	★	★	★	★	★	★	★	★	★

### Meta-analysis results

Crude accumulated incidence of POP was 4.8% (1768/35,362) with an accumulated incidence of 7.8%. (95% CI: 0.061–0.094; I^2^ = 94%). Heterogeneity could not be resolved using sensitivity analyses. Potential risk factors were classified into four categories: basic demographic predictors, medical comorbidity predictors, surgical characteristic predictors, and baseline laboratory predictors. Detailed results for each factor are presented in Tables 3 and 4.

**Table 3:** Pooled risk of demographic characteristics and comorbidities for postoperative pneumonia following hip fracture surgery

	No. of studies	OR or SMD^a^	LL 95% CI	UL 95% CI	*p* value	Heterogeneity (%)	Analysis model
Age	5	0.50^a^	0.10	0.90	0.01	90	Random
Male	10	1.50	1.12	2.01	< 0.01	72	Random
BMI	5	-0.32^a^	−0.90	0.25	0.27	97	Random
Dependent functional status	2	1.87	0.84	4.13	0.12	88	Random
ASA scale ≥3	4	3.17	1.25	8.05	0.02	90	Random
Smoking	6	1.15	0.82	1.60	0.43	0	Fixed
Anemia	3	1.55	1.16	2.08	< 0.01	85	Random
Hypertension	7	1.08	0.89	1.30	0.45	0	Fixed
Diabetes mellitus	9	1.11	0.91	1.37	0.30	7	Fixed
COPD	8	2.05	1.43	2.94	< 0.01	52	Random
Coronary heart disease	6	1.82	1.27	2.60	< 0.01	56	Random
Arrhythmia	4	1.49	1.04	2.15	0.03	0	Fixed
Congestive heart failure	3	1.41	1.14	1.75	< 0.01	5	Fixed
Chronic kidney disease	4	2.09	1.28	3.41	< 0.01	0	Fixed
Cerebrovascular accident	6	2.14	1.60	2.85	< 0.01	22	Fixed
Dementia	3	2.03	0.87	4.71	0.10	59	Random
Cancer	3	1.56	0.93	2.63	0.09	23	Fixed

*OR* Odds ratio, *SMD* Standardized mean difference, *LL* Lower limit, *CI* Confidence interval, *UL* Upper limit, *ASA* American Society of Anesthesiologists physical status, *COPD* Chronic obstructive pulmonary disease

^a^Results of pooled standardized mean difference

**Table 4:** Pooled risk of baseline laboratory data and surgical characteristics of pneumonia following hip fracture surgery

	No. of studies	OR or SMD^a^	LL 95% CI	UL 95% CI	*p* value	Heterogeneity (%)	Analysis model
Hemoglobin	5	−0.14^a^	−0.25	−0.03	0.01	46	Fixed
Albumin	4	−0.97^a^	−1.54	−0.41	< 0.01	95	Random
Blood urea nitrogen	3	0.20^a^	0.03	0.37	0.02	35	Fixed
Creatinine	5	0.22^a^	−0.01	0.46	0.06	76	Random
Aspartate aminotransferase	3	0.00^a^	−0.17	0.17	0.99	27	Fixed
Alanine aminotransferase	3	0.27^a^	0.10	0.44	< 0.01	0	Fixed
Total bilirubin	2	−0.05^a^	−0.24	0.14	0.62	0	Fixed
Arterial O_2_ pressure	2	−0.49^a^	−0.71	− 0.27	< 0.01	0	Fixed
Arterial CO_2_ pressure	2	0.04^a^	−0.18	0.26	0.70	9	Fixed
Intertrochanteric fracture	3	0.90	0.64	1.27	0.55	0	Fixed
Arthroplasty	7	1.24	0.78	1.97	0.35	64	Random
Delayed surgery of over 48 h	3	3.74	2.40	5.85	< 0.01	0	Fixed
Time from injury to surgery	4	0.39^a^	−0.02	0.79	0.06	94	Random
General anesthesia	5	0.99	0.76	1.30	0.95	44	Fixed
Surgical duration	5	−0.01^a^	−0.11	0.10	0.91	0	Fixed
Intraoperative blood loss volume	3	−0.01^a^	− 0.17	0.15	0.90	0	Fixed
Perioperative transfusion rate	2	0.98	0.68	1.40	0.91	0	Fixed

*OR* Odds ratio, *SMD* Standardized mean difference, *LL* Lower limit, *CI* Confidence interval, *UL* Upper limit

^a^Results of pooled standardized mean difference

### Basic demographic predictors

**Fig. 2:**
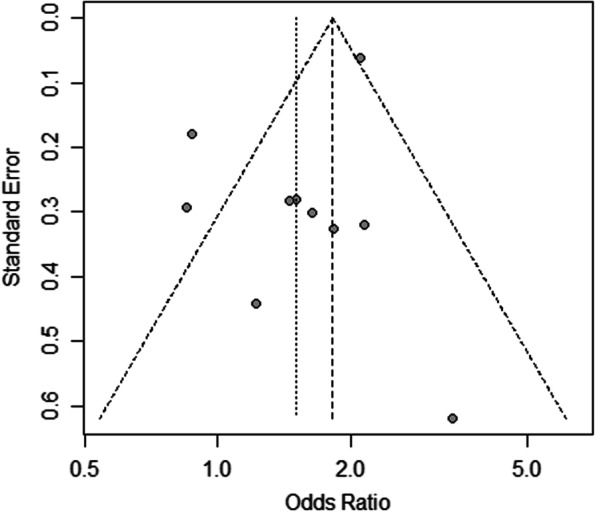
Funnel plot of sex (male) between postoperative pneumonia and no postoperative pneumonia groups

Advanced age (SMD: 0.50; 95% CI: 0.108–0.90; *p* = 0.01; I^2^ = 90%), male sex (OR: 1.50; 95% CI: 1.12–2.01; *p* <  0.01; I^2^ = 72%), and the American Society of Anesthesiologists physical status (ASA) scale ≥3 (OR: 3.17; 95% CI: 1.25–8.05; *p* = 0.02; I^2^ = 90%) were significantly associated with a high risk of POP (Table 3). Significant heterogeneity was found for the pooled results of advanced age, male sex, body mass index, and ASA scale. After sensitivity analyses, heterogeneity was resolved, and the significance did not change (Additional file 1). A funnel plot of sex was symmetrical and suggested a low risk of publication bias (Fig. 2).

### Medical comorbidity predictors

Patients with anemia (OR: 1.55; 95% CI: 1.16–2.08; *p* <  0.01; I^2^ = 85%), chronic obstructive pulmonary disease (COPD) (OR: 2.05; 95% CI: 1.43–2.94; *p* <  0.01; I^2^ = 52%), coronary heart disease (OR: 1.82; 95% CI: 1.27–2.60; *p* < 0.01; I^2^ = 56%), arrhythmia (OR: 1.49; 95% CI: 1.04–2.15; *p* = 0.03; I^2^ = 0%), congestive heart failure (OR: 1.41; 95% CI: 1.14–1.75; *p* < 0.01, I^2^ = 5%), chronic kidney disease (OR: 2.09; 95% CI: 1.28–3.41; *p* < 0.01; I^2^ = 0%), and cerebrovascular accident (OR: 2.14; 95% CI: 1.60–2.85; *p* < 0.01; I^2^ = 22%) were more likely to develop POP after hip fracture surgery (Table 3). Significant heterogeneity was found for anemia, COPD, coronary heart disease, and dementia. After sensitivity analyses, heterogeneity was resolved, and the significance did not change (Additional file 1).

### Baseline laboratory predictors

Lower preoperative hemoglobin (SMD: -0.14; 95% CI: − 0.25 to − 0.03; *p* = 0.01; I^2^ = 46%), lower preoperative serum albumin (ALB) (SMD: -0.97; 95% CI: − 1.54 to − 0.41; *p* < 0.01; I^2^ = 95%), higher preoperative blood urea nitrogen (BUN) (SMD: 0.20; 95% CI: 0.03–0.37; *p* = 0.02; I^2^ = 35%), higher preoperative alanine aminotransferase (SMD: 0.27; 95% CI: 0.10–0.44; *p* < 0.01; I^2^ = 0%), and lower partial pressure of oxygen in arterial blood (SMD: -0.49; 95% CI: − 0.71–-0.27; *p* < 0.01; I^2^ = 0%) indicated an increased risk of POP (Table 4). Significant heterogeneity was observed in the meta-analysis of ALB and creatinine levels. After sensitivity analyses, heterogeneity was resolved for the results of serum creatinine levels without changing the significance (Additional file 1). However, sensitivity analyses could not determine an influential study with high heterogeneity in the ALB level.

### Surgical characteristic predictors

Patients who underwent hip fracture surgery that was delayed for > 48 h from admission or injury had a significantly higher risk of developing POP (OR: 3.74; 95% CI: 2.40–5.85; *p* < 0.01; I^2^ = 0%) (Table 4). Significant heterogeneity was found for surgery type (arthroplasty vs. osteosynthesis) and the time from injury to surgery. After sensitivity analyses, the heterogeneity was resolved, and the intergroup difference in time from injury to surgery was significant (SMD: 0.13; 95% CI: 0.08–0.17; *p* < 0.01; I^2^ = 0%) (Additional file 1).

## Discussion

The present study extensively reviewed and summarized the predictors of POP in patients undergoing hip fracture surgery. A total of 34 predictors were available for meta-analysis, of which 15 predictors, namely, male sex, advanced age, ASA scale ≥3, anemia, COPD, coronary heart disease, arrhythmia, congestive heart failure, chronic kidney disease, cerebrovascular accident, time from injury to surgery, delayed surgery > 48 h after admission or injury, lower preoperative hemoglobin and ALB levels, lower partial pressure of oxygen in arterial blood, and higher BUN and alanine aminotransferase levels, were statistically significant.

POP occurs frequently in patients undergoing hip fracture surgery, particularly in older patients. Results of this meta-analysis revealed that the overall prevalence of POP was 4.8%, which was comparable to the previously reported range of 4.1–15.3% in patients with hip fracture [7–9, 11]. POP is closely associated with prolonged hospital stay and significantly increased mortality [5–8]. It is directly associated with patient prognosis. Therefore, identification and medical optimization of high-risk patients associated with these risk factors are increasingly important.

Advanced age and male sex have long been associated with adverse postoperative morbidities, including POP, in non-cardiac and orthopedic surgeries [20–23]. Airway inflammation and pneumonia increase with age because of swallowing and immune dysfunctions [24–26]. In addition, impaired spirometric lung age, which is correlated with advanced chronological age, is a well-known risk factor for POP [27]. Furthermore, male patients might have more extensive smoking histories, which can modify lung cell biology and impair mucociliary clearance by the increased number of abnormal cilia. In the same context as impaired lung function, the present study found that patients with lower partial pressure of oxygen in arterial blood were more susceptible to POP development.

In terms of basic demographic data predictors, this meta-analysis also found that ASA scale ≥3 was a significant risk factor for POP following hip fracture surgery, consistent with results of previous studies [28, 29]. Therefore, it is needed to give more attention to monitor elderly male patients, particularly those with current status of smoking, dependent functional status, and higher ASA scale, so that early detection could be achieved and prevention strategies could be implemented to reduce POP incidence.

The presence of medical comorbidities has a significant impact in the development of POP after hip fracture surgery. The present study found that anemia, COPD, coronary heart disease, arrhythmia, congestive heart failure, chronic kidney disease, and cerebrovascular accidents were significant risk factors for POP. In particular, comorbid COPD dramatically increases the risk of POP development in patients undergoing hip fracture surgery. COPD is a common condition in elderly patients with hip fractures, and is associated with increased risk of death and postoperative complications [30, 31]. Patients with COPD are in a state of chronic systemic/vascular inflammation and immune system derangements with upregulated C-reactive protein and increased production of inflammatory cytokines and tissue factors [32–34]. Additionally, limited gas exchange and impaired mucociliary clearance of pathogens can predispose patients with COPD to postoperative pulmonary complications [35, 36]. Targeted interventions to reduce the risk of pneumonia are essential in patients with COPD. Potential interventions for COPD include the use of incentive spirometry, elevation of the head of the bed, early ambulation with pain control, and institution of oral hygiene with chlorhexidine [37].

Previous evidence has suggested that anemia is a significant risk factor for postoperative complications, including POP and increased mortality [38, 39]. Consistent with previous studies, the present study showed that patients with comorbid anemia had an increased risk of POP. In the same context, the pooled results showed an increased risk of POP in patients with lower baseline hemoglobin levels. Thus, medical care in the perioperative period, including patient blood management, should be optimized in patients with comorbid anemia to decrease complications, including POP following hip fracture surgery [40].

Evidence suggests that pneumonia is associated with various medical comorbidities, including coronary heart disease, arrhythmia, congestive heart failure, and chronic kidney disease [41–46]. Cerebrovascular accidents are well-known risk factors for dysphagia and pneumonia [8, 47, 48]. Consistent with previous evidence, the pooled results of the present study showed that coronary heart disease, arrhythmia, congestive heart failure, chronic kidney disease, and cerebrovascular accident were significant risk factors for POP following hip fracture surgery. Generally, co-existing medical morbidities are unmodifiable. However, clinicians should have detailed information on coexisting diseases to assess the risk of POP and identify high-risk patients to apply preventive strategies.

Measurement of ALB level can provide an index of severity of protein-energy malnutrition in patients with hip fractures [49]. Preoperative hypoalbuminemia is a well-described risk factor for perioperative morbidity and mortality in patients undergoing orthopedic surgery [50]. In addition, BUN level is frequently elevated in patients with pneumonia because of hydration and increased reabsorption of urea by the kidneys [51, 52]. An elevated BUN/ALB level has also been reported as an independent predictor of mortality and pneumonia severity [51, 53]. Abnormal liver function test results are common in patients with pneumonia. Patients with low ALB or elevated alanine aminotransferase levels show increased mortality and length of stay [54]. Several lines of evidence suggest that the lung liver axis is characterized by a shared and prominent feature of pneumonia with a hepatic acute-phase response [55, 56]. In the same context, the present study found that lower ALB, higher BUN, and higher alanine aminotransferase levels as baseline laboratory predictors were associated with POP development.

Importantly, the present study also found that the time from injury to surgery and delayed surgery for over 48 h after admission or injury were significantly associated with the development of POP. The impact of delays in hip fracture surgery on postoperative complications and mortality has been the object of scientific discussion. Most studies have shown that delays in surgery can lead to worse outcomes, such as mortality, pain, complications, and length of stay [57–62]. Therefore, the international clinical practice guidelines recommend early hip surgery within 48 h of admission, if possible [63].

This study has several strengths. This systematic review and meta-analysis is the first to investigate risk factors for POP in patients undergoing hip fracture surgery. In addition, this meta-analysis was based on the most recent studies published within the last 5 years. Nevertheless, this study has several limitations. First, only retrospective studies with low levels of evidence were included. A general limitation of meta-analyses of observational studies is that the result may be a precise, but biased estimate due to inherent biases and confounding in the original studies. We assessed carefully the quality of the component studies and performed sensitivity analyses excluding studies with a high risk of bias. Second, some of our findings showed a significant heterogeneity and require careful interpretation. However, after sensitivity analyses, the heterogeneity was resolved (I^2^ < 50%) for most results, except for some variables, such as alcohol consumption, ALB level, and the time from injury to surgery. Third, the small sample size might limit the generalizability of the results. Well-designed studies with a large sample and high quality are required in the future.

## Conclusions

This study summarizes numerous predictors of POP in patients undergoing hip fracture surgery. The results can be used to predict the risk of POP development after hip fracture surgery and also provide foundation for future studies. Advanced age, male sex, anemia, diabetes, COPD, coronary heart disease, arrhythmia, congestive heart failure, chronic kidney disease, cerebrovascular accident, surgery over 48 h after injury or admission, lower preoperative serum hemoglobin or ALB levels, lower partial pressure of oxygen in arterial blood, and higher BUN or alanine aminotransferase levels might contribute to the development of POP after hip fracture surgery.

## Supplementary Information

**Additional file 1.** Results of sensitive analysis for variables.

## Data Availability

All data generated or analyzed during this study are included in this published article and its additional information files.

## Abbreviations

POP: Postoperative pneumonia
PRISMA: Preferred Reporting Items for Systematic Reviews and Meta-Analyses
SMD: Standardized mean difference
OR: Odds ratio
CI: Confidence interval
NOS: Newcastle–Ottawa assessment scale
ASA: American Society of Anesthesiologists physical status
COPD: Chronic obstructive pulmonary disease
