# Supplementary material for: Risk factors for postoperative pneumonia in patients undergoing hip fracture surgery: a systematic review and meta-analysis
Source: BMC Musculoskelet Disord. 2022 Jun 8;23:553. doi: 10.1186/s12891-022-05497-1 (PMC9174025; doi:10.1186/s12891-022-05497-1)
Supplement: Supplementary file 1 — Additional file 1. Results of sensitive analysis for variables. [file 12891_2022_5497_MOESM1_ESM.docx]

Additional file 1: Results of sensitive analysis for variables

| Variables | OR or SMD and corresponding 95% CI (original) | *p* value for overall effect | I^2^ | Outlier study excluded | OR or SMD and corresponding 95% CI (afterwards) | *p* value for overall effect | I^2^ |
| --- | --- | --- | --- | --- | --- | --- | --- |
| Age | 0.50 (0.10-0.90) | 0.01 | 92% | Wang 2020  Wang 2020 | 0.51 (0.35-0.67) | <0.01 | 0% |
| Male sex | 1.50 (1.12-2.01) | <0.01 | 72% | Bohl 2018  Xiang 2020 | 1.51 (1.19-1.91) | <0.01 | 7% |
| BMI | -0.32 (-0.90-0.25)* | 0.27 | 97% | Xiang 2020 | -0.08 (0.21-0.05) | 0.23 | 0% |
| ASA scale | 3.17 (1.25-8.05) | 0.02 | 90% | Xiang 2020 | 2.22 (1.51-3.28) | <0.01 | 33% |
| Anemia | 1.55 (1.16-2.08) | <0.01 | 85% | Lv 2016 | 1.22 (1.08-1.37) | <0.01 | 0% |
| COPD | 2.05 (1.43-2.94) | <0.01 | 52% | Xiang 2020 | 1.90 (1.67-2.15) | <0.01 | 31% |
| Coronary heart disease | 1.82 (1.27-2.60) | <0.01 | 56% | Zhao 2020  Ji 2021 | 1.82 (1.35-2.44) | <0.01 | 11% |
| Dementia | 2.03 (0.87-4.71) | 0.10 | 59% | Lv 2016 | 1.26 (0.68-2.32) | 0.46 | 0 |
| Creatinine | 0.22 (-0.01-0.46)* | 0.06 | 76% | Wang 2019 | 0.08 (-0.04-0.20) | 0.17 | 29% |
| Arthroplasty | 1.24 (0.78-1.97) | 0.35 | 64% | Lv 2016 | 1.02 (0.77-1.36) | 0.87 | 19% |
| Time from injury to surgery | 0.39 (-0.02-0.79)* | 0.06 | 94% | Wang 2019 | 0.13 (0.08-0.17) | <0.01 | 0% |

OR, odds ratio; SMD, standardized mean difference; CI, confidence interval; ASA, American Society of Anesthesiologists physical status; COPD, chronic obstructive pulmonary disease

* Results of pooled standardized mean difference
